# Neomorphic DNA-binding enables tumor-specific therapeutic gene expression in fusion-addicted childhood sarcoma

**DOI:** 10.1186/s12943-022-01641-6

**Published:** 2022-10-13

**Authors:** Tilman L. B. Hölting, Florencia Cidre-Aranaz, Dana Matzek, Bastian Popper, Severin J. Jacobi, Cornelius M. Funk, Florian H. Geyer, Jing Li, Ignazio Piseddu, Bruno L. Cadilha, Stephan Ledderose, Jennifer Zwilling, Shunya Ohmura, David Anz, Annette Künkele, Frederick Klauschen, Thomas G. P. Grünewald, Maximilian M. L. Knott

**Affiliations:** 1 Max-Eder Research Group for Pediatric Sarcoma Biology, Institute of Pathology, Faculty of Medicine, LMU Munich, Munich, Germany; 2 Hopp Children’s Cancer Center (KiTZ), Heidelberg, Germany; 3 Division of Translational Pediatric Sarcoma Research, German Cancer Research Center (DKFZ), German Cancer Consortium (DKTK), Heidelberg, Germany; 4 Core Facility Animal Models, Biomedical Center, Ludwig-Maximilians-University, Planegg-Martinsried, Germany; 5 Department of General, Visceral and Transplant Surgery, University Hospital, LMU Munich, Munich, Germany; 6 Division of Clinical Pharmacology, Department of Medicine IV, Klinikum der Universität München, Munich, Germany; 7 Department of Medicine II, University Hospital, Ludwig-Maximilians-Universität München, Munich, Germany; 8 Institute of Pathology, Faculty of Medicine, LMU Munich, Munich, Germany; 9 Department of Pediatric Oncology and Hematology, Charité – Universitätsmedizin Berlin, corporate member of Freie Universität Berlin and Humboldt-Universität zu Berlin, Augustenburger Platz 1, 13353 Berlin, Germany; 10 Berlin Institute of Health at Charité – Universitätsmedizin Berlin, Charitéplatz 1, 10117 Berlin, Germany; 11 German Cancer Consortium (DKTK), Berlin, Germany; 12 German Cancer Consortium (DKTK), partner site Munich, Munich, Germany; 13 Institute of Pathology, Heidelberg University Hospital, Heidelberg, Germany

**Keywords:** Ewing sarcoma, Rhabdomyosarcoma, Fusion oncogene, Targeted therapy, Cancer gene therapy, GPR64

## Abstract

Chimeric fusion transcription factors are oncogenic hallmarks of several devastating cancer entities including pediatric sarcomas, such as Ewing sarcoma (EwS) and alveolar rhabdomyosarcoma (ARMS). Despite their exquisite specificity, these driver oncogenes have been considered largely undruggable due to their lack of enzymatic activity.

Here, we show in the EwS model that – capitalizing on neomorphic DNA-binding preferences – the addiction to the respective fusion transcription factor EWSR1-FLI1 can be leveraged to express therapeutic genes.

We genetically engineered a *de novo* enhancer-based, synthetic and highly potent expression cassette that can elicit EWSR1-FLI1-dependent expression of a therapeutic payload as evidenced by episomal and CRISPR-edited genomic reporter assays. Combining in silico screens and immunohistochemistry, we identified GPR64 as a highly specific cell surface antigen for targeted transduction strategies in EwS. Functional experiments demonstrated that anti-GPR64-pseudotyped lentivirus harboring our expression cassette can specifically transduce EwS cells to promote the expression of viral thymidine kinase sensitizing EwS for treatment to otherwise relatively non-toxic (Val)ganciclovir and leading to strong anti-tumorigenic, but no adverse effects in vivo. Further, we prove that similar vector designs can be applied in PAX3-FOXO1-driven ARMS, and to express immunomodulatory cytokines, such as IL-15 and XCL1, in tumor entities typically considered to be immunologically ‘cold’.

Collectively, these results generated in pediatric sarcomas indicate that exploiting, rather than suppressing, the neomorphic functions of chimeric transcription factors may open inroads to innovative and personalized therapies, and that our highly versatile approach may be translatable to other cancers addicted to oncogenic transcription factors with unique DNA-binding properties.

**Supplementary Information:**

The online version contains supplementary material available at 10.1186/s12943-022-01641-6.

## Background

Unlike most malignancies in adults, childhood sarcomas are commonly characterized by a striking paucity of somatic mutations [1]. However, these entities often harbor tumor-defining fusion oncogenes, such as *EWSR1-FLI1* (EF1) in Ewing sarcoma (EwS) and *PAX3-FOXO1* (P3F1) in alveolar rhabdomyosarcoma (ARMS) acting as potent drivers of malignancy [2, 3]. Both chimeric oncogenes exert their function as aberrant transcription factors equipped with neomorphic features allowing them to bind unique DNA motifs that differ from the binding sites of their parental constituents [4, 5]. For example, EF1 binds to otherwise non-functional GGAA-microsatellites (msats), which are thereby converted into potent *de novo* enhancers [6]. Even though the interaction between EF1 and GGAA-msats is incompletely understood, accumulating evidence suggests that EF1 preferentially binds to GGAA-msats with a specific structure (min. 4 GGAA-repeats; optimal binding at 15–25 GGAA-repeats) [4, 7]. Similarly, P3F1 binds to a highly specific motif (ATTWGTCACGGT), which induces disease-defining, myogenic super enhancers [5, 8]. In both cancer entities, these aberrant DNA binding preferences of the respective chimeric oncoproteins massively deregulate the cellular transcriptome, which promotes their malignant phenotype and oncogene-addiction [5, 9].

Based on the specificity of their interaction with fusion transcription factors and the oncogene-dependency exhibited by the tumors expressing these oncoproteins, we hypothesized that these aberrantly bound neo-enhancers would represent ideal candidates to drive tumor-specific expression of therapeutic genes.

## Results

### Synthetic msat-promoter designs are functional and allow EF1-dependent gene expression

Since the neomorphic DNA-binding preferences of EF1 are very well characterized, we first turned to EwS as a model disease [10–14]. Although reanalysis of publicly available ChIP-seq data generated from two EwS cell lines (A-673, SK-N-MC) demonstrated that most (97.7%) EF1-bound GGAA-msats (defined as at least 4 consecutive GGAA-repeats) were located in intergenic and intronic regions, we identified 4 EF1-bound GGAA-msats located in direct proximity (defined as -1,000 bp to + 100 bp distance) of the transcriptional start site (TSS) of genes annotated in RefSeq (0.4%) (Additional Fig. 1a, Additional Table 1) [4, 6]. Among those, the shortest interval between the identified EF1-bound GGAA-msat and the respective TSS (interval = 49 bp) corresponded to the lncRNA *FEZF1-AS1* (Additional Fig. 1b), which reanalysis of published RNAseq data showed to be significantly downregulated after shRNA-mediated knockdown of EF1 (Additional Fig. 1c). Hence, we assumed that even minimal distance between these EF1-bound neo-enhancers and TSS does not abrogate the transactivating function of EF1.

**Fig. 1:**
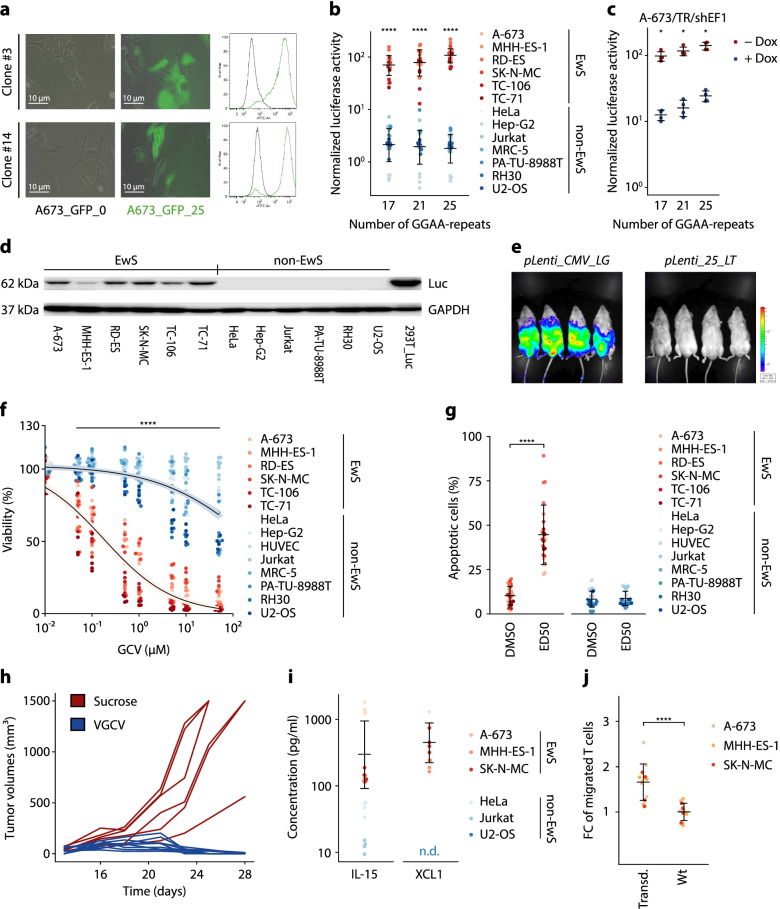
GGAA-msats allow EwS-specific and EF1-dependent gene expression. **a** Fluorescence microscopy images (left) and flow cytometry histograms (right) of A-673 stably transduced with GFP under the control of a minimal promoter and with or without CRISPR/Cas9-mediated knock-in of 25 GGAA-repeats (A673_GFP_25 / A673_GFP_0) in two independent single cell clones. **b** Luciferase reporter assays of indicated EwS and non-EwS cell lines after co-transfection with a reporter plasmid containing the indicated number of GGAA-repeats upstream of the minimal promoter YB-TATA and a constitutively expressed *Renilla*-encoding plasmid. Dots indicate *Firefly* to *Renilla* luminescence ratios normalized to a reporter plasmid without GGAA-repeats for 4 biologically independent experiments. Horizontal bars indicate mean and whiskers standard deviation per group. **c** Luciferase reporter assays of A-673/TR/shEF1 co-transfected with the same plasmids as in Fig. 1b treated with / without Dox. Dots indicate *Firefly* to *Renilla* luminescence ratios normalized to a reporter plasmid without GGAA-repeats for 4 biologically independent experiments. Horizontal bars indicate mean and whiskers standard deviation per group. **d** Detection of Firefly luciferase and GAPDH in protein lysates from EwS and non-EwS cell lines transduced with *pLenti_25_LT_Puro* by Western blot. **e** Bioluminescence measurements (exposure time: 2 min) of NSG mice 14 d after intraperitoneal injection of 1 × 10^7^ TU of VSV-G-pseudotyped *pLenti_25_LT* or *pLenti_CMV_LG* lentiviral particles. **f** Resazurin-based cell viability assay of *pLenti_25_LT_Puro*-transduced and selected EwS and non-EwS cell lines 72 h after GCV addition. Dots indicate relative fluorescence units normalized to vehicle control for 4 biologically independent experiments. Lines show dose-response curves with 95% confidence interval based on a three-parameter log-logistic regression model calculated for EwS or non-EwS cells respectively. **g** Annexin V/PI-staining of *pLenti_25_LT_Puro*-transduced and selected EwS and non-EwS cell lines 72 h after GCV addition. Apoptotic cells were identified as Annexin V (APC) positive cells. Dots indicate the percentage of apoptotic cells for 4 biologically independent experiments. Horizontal bars indicate mean and whiskers the standard deviation. **h** Tumor volumes of *pLenti_25_LT_Puro* pre-transduced subcutaneous xenografts. Valganciclovir (0.5 mg/ml in drinking water enriched with 5% sucrose) or sucrose (5% in drinking water) was administered orally *ad libidum* once the tumor had reached an average diameter of 5 mm. **i** Protein concentrations in conditioned medium of *pLenti_25_IX_Puro*-transduced cell lines measured by ELISA. Dots indicate calculated protein concentration for 4 biologically independent experiments. Horizontal bars indicate mean and whiskers the standard deviation for EwS or non-EwS cell lines. Concentrations below the range of detectability are not depicted in the graph. **j** Transwell Migration Assay using conditioned medium of *pLenti_25_IX_Puro*-transduced and wildtype (wt) cell lines. Migrated CD3^+^ T cells were identified and counted by flow cytometry after 4 h of incubation. Dots indicate the number of migrated CD3^+^ T cells normalized to that in the wt control for each cell line for 4 biologically independent experiments. Horizontal bars indicate mean and whiskers the standard deviation. *P*-values were determined with two-tailed Mann-Whitney test, *: *p* ≤ 0.05, ****: *p* ≤ 0.0001

To test this hypothesis, we generated a EwS reporter cell line, in which we inserted a GGAA-msat directly upstream of a synthetic minimal promoter (YB-TATA) by CRISPR-mediated homology-directed repair (HDR) [15]. Indeed, these clones showed a strong and persistent overexpression of the reporter gene *GFP*, which was not observed in clones lacking the GGAA-msat (Fig. 1a). Thus, the transactivating functionality of EF1 appears to be retained when the GGAA-msat-enhancer is located closely to the respective promoter.

Prior reports have demonstrated that the affinity of EF1 to GGAA-msats, and thereby their enhancer activity, correlates positively with the number of consecutive GGAA-repeats [4, 7]. We therefore tested three different expression cassettes consisting of 17, 21, or 25 GGAA-repeats cloned directly upstream of YB-TATA in a dual luciferase reporter assay in 6 EwS cell lines (including TC-106, a cell line harboring the less common *EWSR1-ERG* fusion oncogene, which is structurally and functionally similar to EF1) and 7 control cell lines, comprising 7 different non-EwS cancer entities or tissue types [1, 16]. Excitingly, we observed a very strong and length-dependent induction by the evaluated GGAA-msats in all tested EwS cell lines whereas there was only minimal induction of reporter activity in the transfected control cell lines (Fig. 1b). To further assess the EF1-dependency of *Firefly luciferase* expression, we repeated these reporter assays in a EwS cell line harboring a doxycycline-inducible shRNA targeting EF1 (A-673/TR/shEF1) or a control shRNA (A-673/TR/shCtrl). Strikingly, conditional knockdown of EF1 dramatically reduced the reporter signal, which was not observed in cells expressing a non-targeting control shRNA (Fig. 1c, Additional Fig. 1d-e). Conversely, ectopic expression of *EF1* in non-EwS osteosarcoma and rhabdomyosarcoma cells (U2-OS, RH30) transfected with the GGAA-msat containing luciferase reporter vector induced the reporter signal while expression of a mutant *EF1* lacking its DNA-binding capacity (EF1_mut R2L2 (ΔEF1) [17]) showed no relevant induction (Additional Fig. 1f). To control for EF1-independent variance in transcriptional activity, we also tested the constitutive promoter of the human elongation factor 1-alpha gene (*EEF1A1*) in transfection-based luciferase assays. In sharp contrast to our newly designed expression cassette, control cell lines showed similar luciferase activity as EwS cell lines when a constitutive promoter was used (Additional Fig. 1g). These results reveal that functional EF1 (or EWSR1-ERG) is necessary for induction of this expression cassette in an episomal setting and demonstrate its superiority over traditional expression systems.

Based on these data, we reasoned that combining a 25 GGAA-repeat element and a minimal promoter could serve as a backbone to mediate the EF1-dependent expression of any therapeutic gene for targeted therapy of EwS.

To test this hypothesis in vitro, we generated a lentiviral transfer plasmid (*pLenti_25_LT_Puro*), containing these regulatory elements followed by the gene encoding a modified *Herpes simplex virus* thymidine kinase *(HSV-TK SR39*) coupled with a *Firefly luciferase* by a P2A linker peptide [18]. We chose *HSV-TK* as a first candidate gene due to its well characterized phenotype and clinical use as suicide-gene in CAR T cell-based therapies [19]. Next, EwS and non-EwS control cell lines were transduced using this vector or an identical control vector lacking the 25 GGAA-repeats (*pLenti_0_LT_Puro)*. Successfully transduced cell lines were selected by puromycin and subjected to reverse transcription qPCR analysis for induction of *HSV-TK* transcription. EwS cell lines showed a significant induction of *HSV-TK* using the *pLenti_25_LT_Puro* vector compared to the control vector without the GGAA-repeats (*pLenti_0_LT_Puro*), whereas in the non-EwS control cell lines the expression levels were similar for both vectors (Additional Fig. 1h). In agreement with these findings at the mRNA level, immunoblotting confirmed that transgenes encoded by *pLenti_25_LT_Puro* were only detectable in EwS cells but not in non-EwS cells at the protein level (Fig. 1d). As single cell lines do not reflect the complexity of tissues or organisms, we sought to evaluate the specificity of our expression cassette in vivo. To this end, we generated a transfer plasmid (*pLenti_25_LT*) similar to *pLenti_25_LT_Puro* but lacking the puromycin resistance cassette and intraperitoneally injected 1 × 10^7^ TU of VSV-G pseudotyped lentiviral particles carrying either *pLenti_25_LT* or a CMV-driven *luciferase* (*pLenti_CMV_LG*). Excitingly, no luciferase signal was detected in the *pLenti_25_LT* group, whereas strong luciferase signal was obtained in the thoracoabdominal region of *pLenti_CMV_LG-*transduced animals (Fig. 1e). To exclude differences in transduction efficiency, we harvested the organs and found comparable copy numbers of both vectors by genomic qPCR (Additional Fig. 1i).

These results predicted that EwS cells transduced with this vector should react with increased sensitivity to treatment with ganciclovir (GCV) compared to non-EwS cells. Indeed, when assessing cell viability after GCV treatment in resazurin-based viability assays, EwS cell lines transduced with *pLenti_25_LT_Puro* showed ~ 100-fold lower effective dose 50 (ED50) concentrations than control cell lines (Fig. 1f). To correct for transgene independent differences in GCV-sensitivity, we also included *pLenti_0_LT_Puro-*transduced cell lines. Notably, GCV-toxicity was only induced in EwS cell lines, whereas control cell lines showed similar ED50 values for both vectors (Additional Fig. 1j). In line with these observations, GCV-treatment using the average ED50 values of EwS cell lines (0.4 µM) induced extensive cell death in EwS cells but not in non-EwS controls as evidenced by Annexin V/Propidium iodide staining and flow cytometric analysis (Fig. 1g). Strikingly, upon systemic treatment with Valganciclovir (VGCV) *per os*, complete tumor regression was observed in a pre-transduced EwS xenograft model (RD-ES) (Fig. 1h), without any detectable adverse effects, such as differences in body weight (Additional Fig. 1k) or histomorphological changes in inner organs (not shown). Taken together, these in vitro and in vivo data generated in EwS models suggested that the DNA-binding preferences mediated by neomorphic functions of fusion transcription factors could be exploited to deliver a therapeutic payload with high specificity and fidelity.

To demonstrate the versatility of our expression cassette for different therapeutic approaches, we explored its suitability for tumor-specific overexpression of cytokines that may sensitize EwS for immunotherapeutic strategies, such as chimeric antigen receptor (CAR) T cell therapy. Thus, we replaced the *HSV-TK* coupled to *Firefly luciferase* in *pLenti_25_LT_Puro* by the cytokines *IL-15* and *XCL1* coupled by a P2A-linker peptide (*pLenti_25_IX_Puro*). Both cytokines are known to confer a strong activating (IL-15) and chemoattractive (XCL1) effect on T cells [20–22]. Similar to our findings with *HSV-TK*, ELISA demonstrated that EwS cells, but not non-EwS control cells transduced with this new vector secreted these cytokines at relevant levels (Fig. 1i). Consistently, conditioned medium of EwS cells transduced with *pLenti_25_IX_Puro* was able to stimulate the migratory activity of T cells (Fig. 1j). Taken together, these in vitro data suggested that our expression cassette can be used as a flexible tool for EwS-specific expression of therapeutically exploitable genes.

### GPR64 is a promising target for targeted gene delivery in EwS

**Fig. 2:**
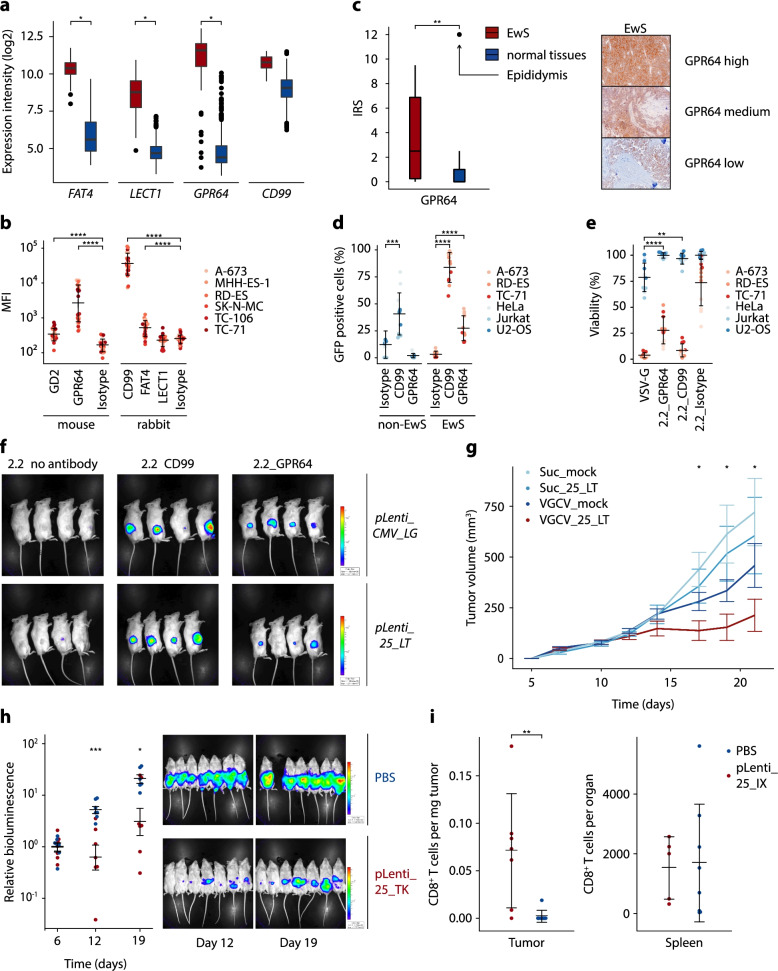
Combination of EwS-specific expression cassette and targeted gene delivery confers strong therapeutic effects in vivo. **a** mRNA log2 expression intensities of *GPR64*, *FAT4*, *LECT1*, and *CD99* from publicly available microarray data of EwS (*n* = 50) and normal tissues (*n* = 928, comprising 70 different tissue types). Data are presented as boxplots with the horizontal line representing the median, the box the interquartile range (IQR) and the whiskers 1.5×IQR of the expression intensity. **b** Validation of surface expression of GD2, GPR64, CD99, FAT4 and LECT1 by antibody staining and flow cytometry. Isotype controls for both antibody host species were included separately. Dots indicate mean fluorescent intensity (MFI) for 4 independent experiments. Mean and standard deviation per group are depicted as horizontal bars and whiskers. **c** IRS (immunoreactive score) of GPR64 in immunohistochemistry of primary EwS tumors and relevant normal tissues. Representative EwS samples with high, medium and low GPR64 expression are shown aside. **d** Flow cytometry analysis of EwS and non-EwS cell lines after transduction with GPR64-targeting, *GFP*-encoding lentiviruses. CD99 and isotype- targeting lentivirus was used as positive and negative control. Dots indicate percentage of GFP positive cells determined by flow cytometry of 4 biologically independent experiments. Horizontal bars and whiskers represent mean and standard deviation per group. **e** Resazurin-based cell viability assay of EwS and non-EwS cell lines treated with GCV (20 µM) or DMSO vehicle control 24 h after GPR64-targeted transduction with *pLenti_25_LT.* Readout was performed 72 h after GCV addition. CD99-targeting lentiviruses, non-targeting lentiviruses (isotype) and VSV-G pseudotyped lentiviruses were included as controls. Dots indicate cell viability relative to that of vehicle control for 4 biologically independent experiments. Mean standard deviation per group are represented by horizontal bars and whiskers. **f** Bioluminescence measurements (exposure time: 20 s) of NSG mice bearing subcutaneous RD-ES xenografts 14 d after a single intratumoral injection of 0.5 × 10^6^ TU of *pLenti_25_LT* or *pLenti_CMV_LG* lentiviral particles pseudotyped with 2.2. GPR64- or CD99-targeting antibodies were used to coat 2.2 pseudotyped viruses. 2.2 pseudotyped viruses without antibodies were included as negative control. **g** Tumor volumes of A-673 subcutaneous xenografts treated with GPR64-targeting *pLenti_25_LT* or *pLenti_CMV_LG* (mock) lentiviruses. Valganciclovir (VGCV, 0.5 mg/ml in drinking water enriched with 5% sucrose) or sucrose (5% in drinking water) was administered orally *ad libidum* once the tumor had reached an average diameter of 5 mm. Lentiviruses were intratumorally injected twice per week starting from day 7. Data are shown as mean tumor volume and SEM of 6–7 mice per treatment condition. *P*-values were determined by one-tailed Mann-Whitney test. **h** Relative bioluminescence (right) and bioluminescent images (left) of NSG mice after intraperitoneal tumor inoculation with *Firefly* luciferase-expressing A-673. 3 days after tumor injection mice were randomized and repeatedly received either GPR64-directed 2.2. pseudotyped lentivirus (*pLenti_25_TK*) or PBS by intraperitoneal injection. VGCV was orally administered in both groups 3 days after the first virus injection. The representative bioluminescent pictures show both groups 12 and 19 days after tumor inoculation. Dots indicate bioluminescence signal relative the mean measured on of VGCV initiation (day 6) for 6–7 mice per group. Horizontal bars indicate mean and whiskers SEM per group. *P* values were determined by one-tailed Mann-Whitney test. **i** CD8^+^ T cell count per mg of tumor tissue and absolute CD8^+^ T cell count per spleen 5 days after human T cell transfer into mice bearing subcutaneous A-673 xenografts treated with GPR64-coated lentiviral particles (*pLenti_25_IX*) or PBS. Where not indicated otherwise, *P*-values were determined with two-tailed Mann-Whitney test, *: *p* ≤ 0.05, **: *p* ≤ 0.01, ***: *p* ≤ 0.001, ****: *p* ≤ 0.0001

Having successfully designed and characterized a highly specific expression cassette, we sought to develop a suitable delivery strategy for therapeutic purposes in vivo. To increase the specificity and to enhance the viral load reaching the tumor in a therapeutic setting, we sought to combine the EwS-specific expression system with a EwS-specific transduction method, which should greatly diminish the amount of vector being lost by transducing non-target cells. Pseudotyping lentiviral particles with a modified and optimized Sindbis glycoprotein (*2.2*) containing Fc region-binding sites of protein A has been shown to allow antibody-mediated transduction in vivo [23, 24]. As previously described, 2.2-pseudotyped viral particles are devoid of any natural tropism and enable highly specific viral transduction by E1-mediated fusion of envelope and cell membrane only in presence of target cell-specific antibodies [23, 24]. While in principle CD99 would constitute a highly expressed surface protein in EwS, its ubiquitous expression in normal tissues renders this protein unsuitable for such an approach [25]. To identify EwS-specific candidate surface proteins that are highly expressed in EwS but only minimally in normal tissues, we analyzed a previously described set of gene expression microarray data from 50 EwS and 928 normal tissues (comprising 70 tissue types) and identified 36 genes that were significantly overexpressed in EwS compared to any other normal tissue (Additional Table 2) [25]. Of these, 3 genes (*GPR64*, *FAT4* and *LECT1*) encoding cell surface proteins were selected for in vitro analysis based on the availability of commercial monoclonal antibodies targeting their extracellular domains (Fig. 2a, Additional Fig. 2). Indirect antibody staining and flow cytometry analysis confirmed the surface-expression of GPR64 and, to a lesser extent, of FAT4 in 6 EwS cell lines at the protein level (Fig. 2b). Interestingly, the membrane-bound disialoganglioside GD2, which was recently identified as potential target for antibody- or CAR T cell-based therapies, showed only a weak staining signal in the 6 EwS cell lines tested [26]. Thus, due to its higher expression levels, GPR64 was selected for further experiments and its specific expression was confirmed in situ in patient-derived EwS tumor tissue (*n* = 18) and normal tissues (*n* = 29) by immunohistochemistry (Fig. 2c). Notably, apart from the epididymis, only minimal GPR64 expression was found in any other organ whereas the majority of EwS samples showed positive staining in immunohistochemistry (Additional Table 3).

To evaluate the suitability of GPR64 as a candidate for targeted transduction of EwS cells, lentiviral particles were produced using a transfer plasmid containing a GFP reporter expressed by a CMV promoter and the 2.2 packaging plasmid. Next, EwS (A-673, RD-ES, TC-71) cell lines and non-EwS (HeLa, Jurkat, U2-OS) control cell lines were transduced with these vectors combined with either a GPR64 antibody, a CD99 antibody, or an isotype control. Remarkably, flow cytometry analysis showed specific GFP expression of EwS cells when targeting GPR64 while no significant GFP-positivity was seen in control cells or when isotype-coated virus was added (Fig. 2d). In accordance with its ubiquitous expression, CD99-coated viral particles showed non-specific transduction of both EwS and control cell lines.

To assess whether the addition of this transduction-based targeting strategy could further increase the therapeutic specificity of our transcription-based approach, 3 EwS and 3 control cell lines were treated with equal amounts of lentivirus either pseudotyped by VSV-G, or antibody-coated 2.2 (GPR64, CD99 or isotype control) using the aforementioned transfer plasmid *pLenti_25_LT*. Subsequent addition of GCV (20 µM) revealed a significant reduction in GCV sensitivity in non-EwS control cells treated with GPR64-coated viral particles compared to those treated with VSV-G pseudotyped virus, indicating less specific incorporation of VSV-G pseudotyped virus, which underlines the benefit of the additional EwS-specific delivery strategy (Fig. 2e).

Next, we aimed to investigate whether antibody-mediated transduction of EwS cells was also feasible in vivo. To this end, we intratumorally injected 2.2-pseudotyped and antibody-coated lentiviral particles carrying the *Firefly luciferase* transgene under control of our EwS-specific expression cassette (*pLenti_25_LT*) or driven by the ubiquitous CMV promoter (*pLenti_CMV_LG*) into subcutaneous RD-ES xenografts (Fig. 2f). Notably, comparable tumor-derived luminescence was detected when injecting GPR64- or CD99-directed, 2.2-pseudotyped compared to VSV-G-pseudotyped lentiviruses that served as positive control. Moreover, plain, uncoated 2.2-pseudotyped viruses achieved no detectable transduction both in the *pLenti_25_LT* and *pLenti_CMV_LG* group. These results were confirmed in a second cell line (A-673) (Additional Fig. 3a). In sum, these experiments demonstrate the feasibility of antibody-mediated GPR64-targeted transduction of EwS cells in vivo.

**Fig. 3:**
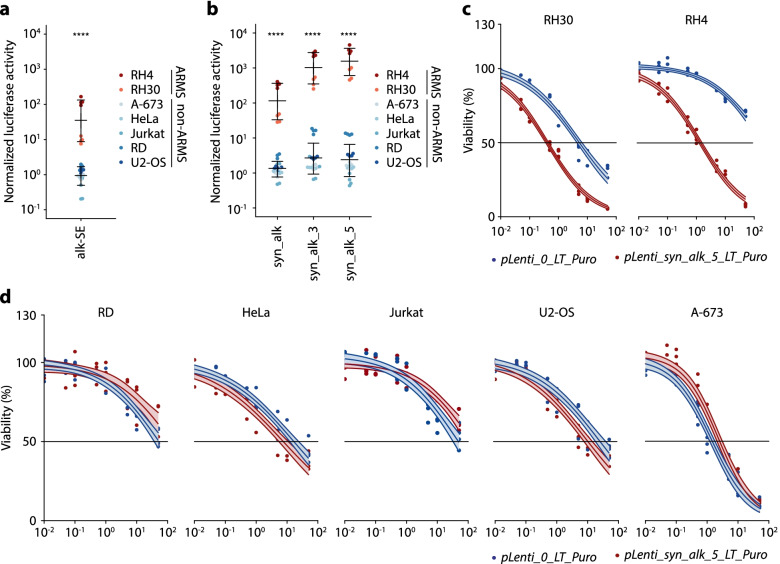
Highly specific, enhancer-based gene expression systems can be designed for other fusion-driven pediatric sarcomas. **a** Luciferase reporter assays of indicated fusion-positive ARMS (RH4 and RH30) and control cell lines after co-transfection with a reporter plasmid containing the alk-SE upstream of the minimal promoter YB-TATA and a constitutively expressed *Renilla* luciferase-encoding plasmid. Dots indicate *Firefly* to *Renilla* luminescence ratios normalized to a reporter plasmid without the alk-SE. Horizontal bars indicate mean and whiskers standard deviation per group. **b** Luciferase reporter assays of the same cell lines as in Fig. 3a after co-transfection with a reporter plasmid containing either syn_alk, syn_alk_3 or syn_alk_5 upstream of the minimal promoter YB-TATA and a constitutively expressed *Renilla* luciferase-encoding plasmid. Dots indicate *Firefly* to *Renilla* luminescence ratios normalized to a reporter plasmid without the alk-SE. Horizontal bars indicate mean and whiskers standard deviation per group. **c** Resazurin-based cell viability assay of *pLenti_syn_alk_5_LT_Puro*- or *pLenti_0_LT_Puro*-transduced and selected P3F1-positive ARMS cell lines 72 h after GCV addition. Dots indicate relative fluorescence units normalized to vehicle control for 4 biologically independent experiments. Lines show dose-response curves with 95% confidence interval based on a three-parameter log-logistic regression model calculated for each cell line. **d** Resazurin-based cell viability assay of P3F1-negative control cell lines transduced as in Fig. 3c. Dots indicate relative fluorescence units normalized to vehicle control for 4 biologically independent experiments. Lines show dose-response curves with 95% confidence interval based on a three-parameter log-logistic regression model calculated for each cell line. *P*-values were determined by two-tailed Mann-Whitney test, ****: *p* ≤ 0.0001

### The combination of EwS‑specific delivery and gene expression improves specific tumor therapy in vivo

Having established both, a EwS-specific expression cassette and delivery strategy, we moved on to combine these two for therapeutic purposes in vivo. Therefore, we subcutaneously inoculated A-673 EwS cells and, once the average tumor diameter had reached 5 mm, intratumorally injected GPR64-directed, 2.2-pseudotyped treatment (*pLenti_25_LT*) or mock (*pLenti_CMV_LG*) virus. Excitingly, upon oral VGCV administration, most tumors in the *pLenti_25_LT*-transduced group showed significant reduction in tumor growth compared to the control groups (Fig. 2g). In a second step, we evaluated the efficacy of our treatment strategy in a more systematic setting by inoculating luciferase-expressing A-673 cells intraperitoneally and repeatedly injecting GPR64-directed, 2.2-pseudotyped lentivirus expressing *HSV-TK* (*pLenti_25_TK*) or PBS (negative control) into the peritoneal cavity 3 days after tumor inoculation. Excitingly, while the control group showed a strong increase of luminescence over time corresponding to strong increase in peritoneal tumor mass, a significantly lower increase in bioluminescent signal was detected in the treatment group (Fig. 2h and Additional Fig. 3b). Next, we set out to assess the efficacy of immunomodulatory strategies using our expression cassette. To this end, we injected GPR64-directed, 2.2-pseudotyped viral particles carrying the transgenes IL-15 and XCL1 coupled by a P2A linker peptide (*pLenti_25_IX*) or PBS (negative control) into subcutaneous A-673 xenografts. Additionally, we transferred 1 × 10^7^ GFP-transduced human T cells by intravenous injection. Notably, 5 days after T cell transfer, we found a significant intratumoral enrichment of human CD8^+^ T cells in transduced mice compared to the untreated (PBS) control (Fig. 2i).

In conclusion, our results indicate that the neomorphic aberrant DNA-binding properties of EF1 enable EwS specific and EF1-dependent expression of therapeutic transgenes in vivo.

### Highly specific, enhancer-based gene expression systems can be designed for other fusion-driven pediatric sarcomas

To investigate whether this principle can be translated to other cancers driven by an oncogenic fusion transcription factor, we extended our analyses to fusion-positive ARMS, which harbors the dominant chimeric P3F1 oncoprotein in more than 50% of cases [27]. P3F1 mediates cell transformation by binding to specific DNA motifs thereby establishing *de novo* super-enhancers (SEs) encompassing known oncogenes, such as *ALK*, causing dysregulating of the transcriptome [5, 8, 28]. Thus, we first cloned a ~ 300 bp DNA segment (chr2:29,657,671–29,657,976; hg38) from the third intron of *ALK*, that has been identified as a strong P3F1-binding site, into a luciferase reporter plasmid upstream of YB-TATA [5]. Similar to our observations made in EwS (Fig. 1b), we found a significant induction of reporter gene expression in fusion-positive ARMS (RH4 and RH30) but not in fusion-negative embryonal rhabdomyosarcoma (RD) or in non-rhabdomyosarcoma control cell lines (U2-OS, HeLa, Jurkat and A-673) (Fig. 3a). Interestingly, Gryder et al. showed that two point mutations of a single P3F1-binding motif, consisting of GTCACGGT, abrogated the transactivating activity of the ALK-SE [5]. To further improve the induction capacity of this construct, we optimized the sequence at this putative P3F1 binding site to completely match the ATTWGTCACGGT motif (syn_alk) as annotated by HOMER motifs, which resulted in improved luciferase signals in fusion positive ARMS cell lines but not in control cell lines (Fig. 3b). Strikingly, the fusion positive ARMS-specific expression induction could be further increased by adding three (syn_alk_3) or five (syn_alk_5) additional ATTWGTCACGGT motifs to the SE sequence (Fig. 3b). In accordance with our previous experiments in EwS, the best performing ARMS-specific expression cassette (containing syn_alk_5) cloned upstream of *HSV-TK* induced GCV sensitivity only in P3F1-positive cells, while control cell lines showed no increase in GCV sensitivity compared to a control promoter containing YB-TATA alone (Fig. 3c-d).

Collectively, these results indicate that, in principle, our approach is translatable to other cancers driven by oncogenic transcription factors with unique DNA-binding properties.

## Conclusion

In summary, our results provide evidence that the unique interaction of oncogenic fusion transcription factors with aberrant binding sites can be used for specific therapeutic gene expression. In sharp contrast to existing strategies for tumor specific gene expression, which usually take advantage of aberrant promoter activation (hTERT [29], AFP [30]), hypoxic conditions in the tumor-microenvironment (HRE [31]) or tissue-of-origin-specific gene expression patterns (PSA [32]), our design relies on the unique and aberrant activity of tumor-defining fusion oncogenes, rather than the physiological action of non-mutated, but aberrantly expressed transcription factors. Moreover, our design exploits the aberrant binding of single *de-novo* motifs (GGAA-mSat in EwS, ATTW**GTCACGGT** in ARMS), while traditionally used promoter sequences usually allow the binding of a plethora of different transcription factors. Indeed, we could not detect any significant off-target activity of our GGAA-mSat-based expression cassette in mice after systemic delivery of VSV-G pseudotyped lentiviral particles, underlining the high specificity of our design.

In this study, we chose *HSV-TK* and immunostimulatory cytokines as tumor-specifically expressed transgenes. While suicide-genes such as *HSV-TK* can eradicate even incompletely transduced tumors by the so-called bystander effect, any off-target expression can confer serious side effects. Similarly, immunotherapies need to be administered carefully due to their high potential to trigger autoimmune reactions against normal tissues [33]. Thus, the expression of transgenes that inactivate tumor-specific (onco)genes or replace mutated tumor suppressor genes might represent an appealing strategy. However, pediatric sarcomas are frequently oligomutated and alterations in oncogenes and tumor suppressor genes usually found in adult cancer entities (e.g., *TP53*, *KRAS*, *PTEN*, and *BRAF*), are exceedingly rare [34]. Moreover, tumorigenic networks are often redundant and the inhibition or reactivation of certain components is therefore unlikely to achieve long-lasting therapeutic effects. Hence, tumor-specific expression of suicide genes and immunostimulatory cytokines represents an efficient and possibly translational strategy for cancer gene therapies especially in oligomutated pediatric tumors.

Apart from a lack of specificity, which our promoter design alleviates, the delivery of the therapeutic transgene to sufficient numbers of cancer cells is another limitation of current cancer gene therapies and may limit their clinical translation. Despite their strength as preclinical model vectors, the integrating and non-replicating nature of lentiviruses, such as the ones employed in this proof-of-concept study, render them less suitable for safe and sufficient gene delivery. However, as the fusion oncogene-based expression systems proposed by us are of a simple architecture and show tumor specificity both in integrating as well as episomal vectors, they could be used in future studies for any therapeutic approach relying on transgene expression in cancer cells, including replicating oncolytic viruses.

## Supplementary Information

**Additional file 1:** Additional Figures 1–4 and Additional Figure Legends.

**Additional file 2:** Additional Tables 1-5.

**Additional file 3:** Additional Methods.

## Data Availability

All ChIP-seq, RNA-seq and Microarray data reanalyzed as part of this study are publicly available under the accession codes listed in Additional Table 5 and the methods section.

## Abbreviations

AFP: Alpha fetoprotein
APC: Allophycocyanin
ARMS: Alveolar rhabdomyosarcoma
CAR: Chimeric antigen
CMV: Cytomegalovirus
DMSO: Dimethyl sulfoxide
Dox: Doxycycline
ED: Effective dose
EF1: EWSR1-FLI1
EwS: Ewing sarcoma
Fc: Fragment crystallizable
FITC: Fluorescein isothiocyanate
GAPDH: Glyceraldehyde-3-Phosphate Dehydrogenase
GCV: Ganciclovir
GFP: Green fluorescent protein
HRE: Hypoxia response element
HSV: Herpes simplex virus
hTERT: human telomerase reverse transcriptase
IRS: Immunoreactive score
KD: Knockdown
LG: Luciferase-P2A-GFP
LT: Luciferase-P2A-thymidine kinase
MFI: Mean fluorescence intensity
P3F1: PAX3-FOXO1
PE: Phycoerythrin
PI: Propidium iodide
PSA: Prostate-specific antigen
Puro: Puromycin
RT: Room temperature
SE: Super-enhancer
TK: Thymidine kinase
TU: Transducing units
VGCV: Valganciclovir
VSV-G: Vesicular stomatitis virus glycoprotein
WT: Wildtype
