# Supplementary material for: Neomorphic DNA-binding enables tumor-specific therapeutic gene expression in fusion-addicted childhood sarcoma
Source: Mol Cancer. 2022 Oct 13;21:199. doi: 10.1186/s12943-022-01641-6 (PMC9558418; doi:10.1186/s12943-022-01641-6)

Additional figure legends

Additional Fig. 1

a Genomic annotation of location of EF1-bound GGAA-msats (min. 4 GGAA-repeats). **b** Epigenetic profile of the *FEZF1-AS1* locus in indicated EwS cells transduced with either a control shRNA (shGFP) or a specific shRNA against *EF1* (shEF1) from published ChIP-seq data for EWSR1-FLI1 and H3K27ac[6]. **c** Volcano plot of published RNA-seq data showing differentially expressed genes (DEGs) after shRNA-mediated *EF1* (shEF1) knockdown compared to a non-targeting shRNA (shGFP). A summary of two cell lines is shown (A-673 and SK-N-MC). *FEZF1-AS1* is depicted in red. **d** Luciferase reporter assays of A-673/TR/shCtrl co-transfected with the same plasmids as in Fig. 1b treated with / without Dox. Dots indicate *Firefly* to *Renilla* luminescence ratios normalized to a reporter plasmid without GGAA-repeats for 4 biologically independent experiments. Horizontal bars indicate mean and whiskers standard deviation per group. **e** Analysis of *EF1* mRNA expression after 72 h of doxycycline treatment compared to untreated controls in A-673/TR/shEF1 cells by RT-qPCR. Dots indicate gene expression relative to untreated controls by determined by the $2^{-\Delta\Delta CT}$ method for 4 independent experiments. Horizontal bars indicate mean and whiskers standard deviation per group. **f** Luciferase reporter assays of non-EwS cell lines RH30 and HeLa co-transfected with the same plasmids as in Fig. 1b and a plasmid expressing EF1 or a defective mutant of EF1 (ΔEF1). Dots indicate *Firefly* to *Renilla* luminescence ratios normalized to a reporter plasmid without GGAA-repeats for 4 biologically independent experiments. Horizontal bars indicate mean and whiskers standard deviation per group. **g** Luciferase reporter assays of indicated EwS and non-EwS cell lines after co-transfection with a reporter plasmid containing either the constitutive promoter of the human elongation factor 1-alpha gene or 25 GGAA repeats upstream of the minimal promoter YB-TATA

and a constitutively expressed *Renilla*-encoding plasmid. Dots indicate *Firefly* to *Renilla* luminescence ratios normalized to a reporter plasmid without GGAA-repeats for 4 biologically independent experiments. Horizontal bars indicate mean and whiskers standard deviation per group. **h** Analysis of *HSV TK* mRNA expression in *pLenti_25_LT_Puro*-transduced and selected EwS and non-EwS cell lines compared to *pLenti_0_LT_Puro*-transduced and selected EwS and non-EwS cell lines. Dots indicate ΔCT values of *TK* compared to *RPLP0*. Horizontal bars indicate mean and whiskers standard deviation per group. **i** Analysis of *WPRE* copy numbers in indicated organs after intrapertioneal injection of VSV-G pseudotyped *pLenti_25_LT* or *pLenti_CMV_LG* lentiviral particles by genomic qPCR. Dots indicate ΔCT values of *WPRE* compared to murine *ACTB* for 4 mice per group. Horizontal bars indicate mean and whiskers standard deviation per group. **j** Resazurin-based cell viability assay of *pLenti_25_LT_Puro*- or *pLenti_0_LT_Puro*-transduced and selected EwS and non-EwS cell lines 72 h after GCV addition. Dots indicate relative fluorescence units normalized to vehicle control for 4 biologically independent experiments. Lines show dose-response curves with 95% confidence interval based on a three-parameter log-logistic regression model calculated for EwS or non-EwS cells respectively. **k** Weight curves of VGCV-(0.5 mg/ml) treated and untreated (sucrose) tumor bearing NSG mice. Lines indicate mean weights of 7-10 mice per group and whiskers standard error of mean.
P-values were determined by two-tailed Mann-Whitney test, *: $p \leq 0.05$, ****: $p \leq 0.0001$.

Additional Fig. 2

mRNA log2 expression intensities of **a** *FAT4*, **b** *LECT1* and **c** *GPR64* from publicly available microarray data of EwS (n = 50) and normal tissues (n = 928, comprising 70 different tissue types). Data are presented as boxplots with the horizontal line representing the median, the box the interquartile range (IQR) and the whiskers 1.5 * IQR of the expression intensity.

Additional Fig. 3

a Bioluminescence measurements (exposure time: 3 sec) of NSG mice bearing subcutaneous A-673 xenografts 14 d after a single intratumoral injection of 0.5×10^6 TU of *pLenti_25_LT* or *pLenti_CMV_LG* lentiviral particles pseudotyped with 2.2. Anti-GPR64 antibody was used to coat 2.2 pseudotyped viruses. 2.2 pseudotyped viruses without antibody were included as negative control. **b** Bioluminescent images of NSG mice (exposure time: 2 sec) after intraperitoneal tumor inoculation with luciferase-expressing A-673. 3 days after tumor injection mice were randomized and repeatedly received either GPR64-directed 2.2. pseudotyped lentivirus (*pLenti_25_TK*) or PBS by intraperitoneal injection. VGCV was orally administered in both groups 3 days after the first virus injection. The representative bioluminescent pictures show both groups 12 and 19 days after tumor inoculation. The timeline below depicts the detailed design of the experiment. **c** $CD4^+$ T cell count per mg of tumor tissue and absolute $CD8^+$ T cell count per spleen 5 days after human T cell transfer into mice bearing subcutaneous A-673 xenografts treated with GPR64-coated lentiviral particles (*pLenti_25_IX*) or PBS. Horizontal bars and whiskers represent mean and standard deviation per group.

Additional Fig. 4

a Representative flow cytometry gating strategy for identification of apoptotic EwS and non-EwS control cell lines after GCV treatment. Density plots show a representative sample of SK-N-MC cells treated with 0.4 µM GCV for 72 h. Annexin V was stained by APC. **b** Representative flow cytometry gating strategy for identification of migrated T cells and counting beads. Density plots show a representative sample of PBMC migrated towards conditioned medium of *pLenti_25_IX_Puro* pre-transduced and selected A-673. CD3 was stained by FITC. Dead cells were excluded by PI staining. **c** Representative flow cytometry gating strategy for identification of

surface antigen expression by indirect staining procedure. Density plots show a representative sample of A-673 cells indirectly stained for GPR64 (APC) after exclusion of dead cells by PI (PE). **d** Representative flow cytometry gating strategy for identification of GFP-transduced EwS and non-EwS control cell lines after antibody-mediated transduction. Density plots show a representative sample of A-673 cells transduced with GPR64-targeting vectors. **e** Representative flow cytometry gating strategy for identification of human T cells isolated from mice tissue stained for CD3 (PacBlue), CD4 (PE), CD8 (APC). Dead cells were excluded by *Zombie Aqua*™ (AmCyan). Density plots show a representative sample of splenic cells.

Additional Fig. 1

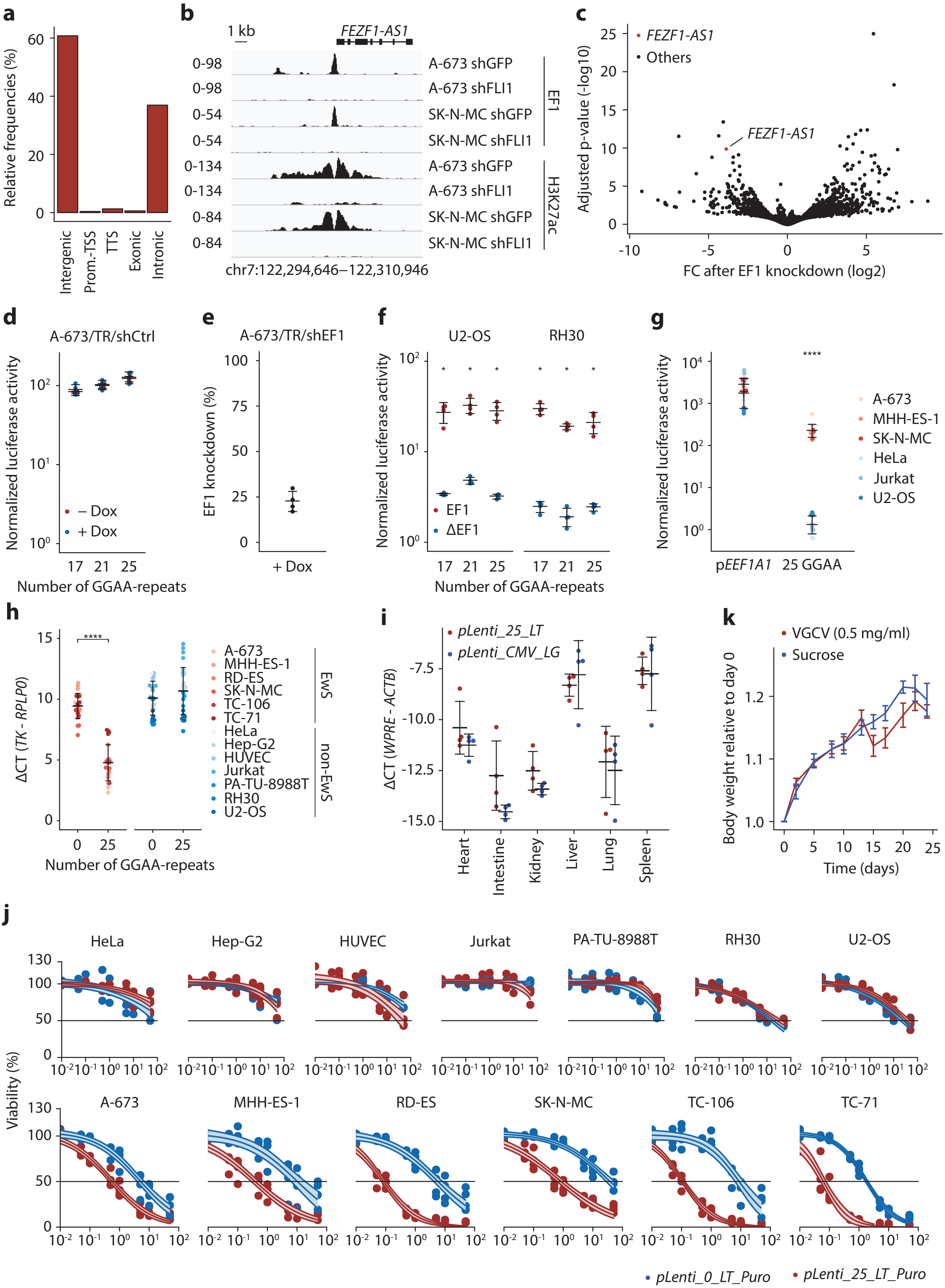

Additional Fig. 2

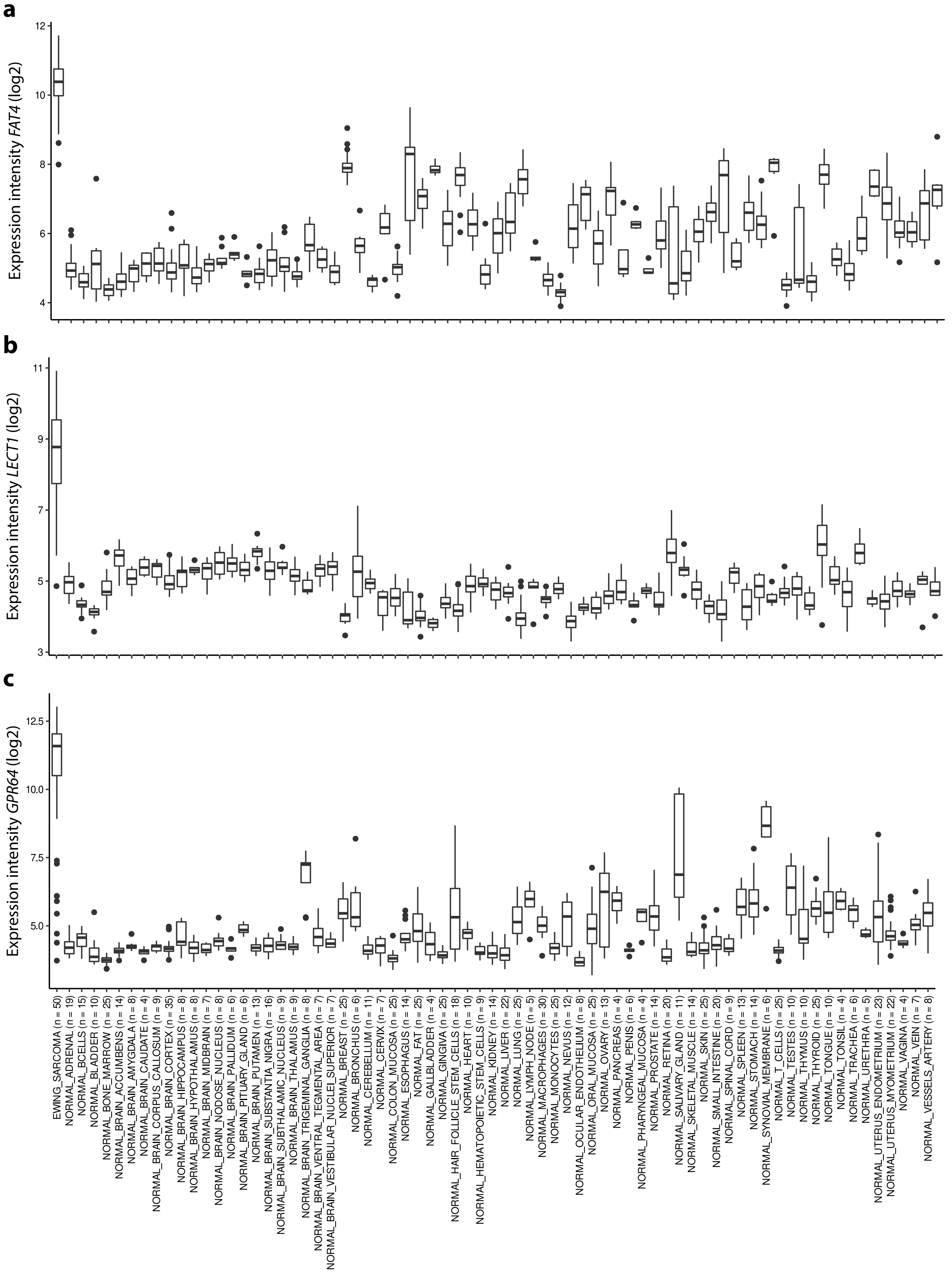

Additional Fig. 3

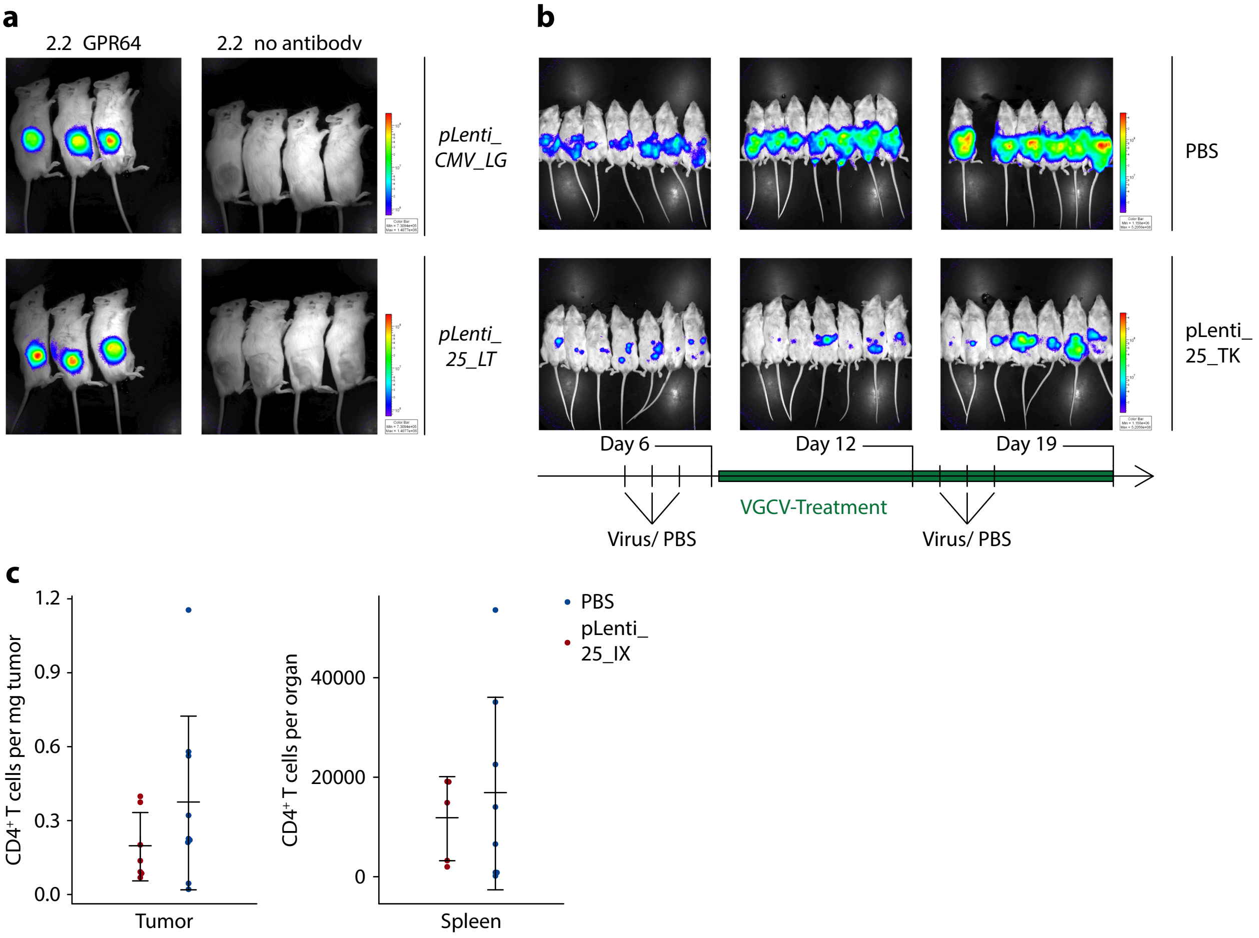

Additional Fig. 4

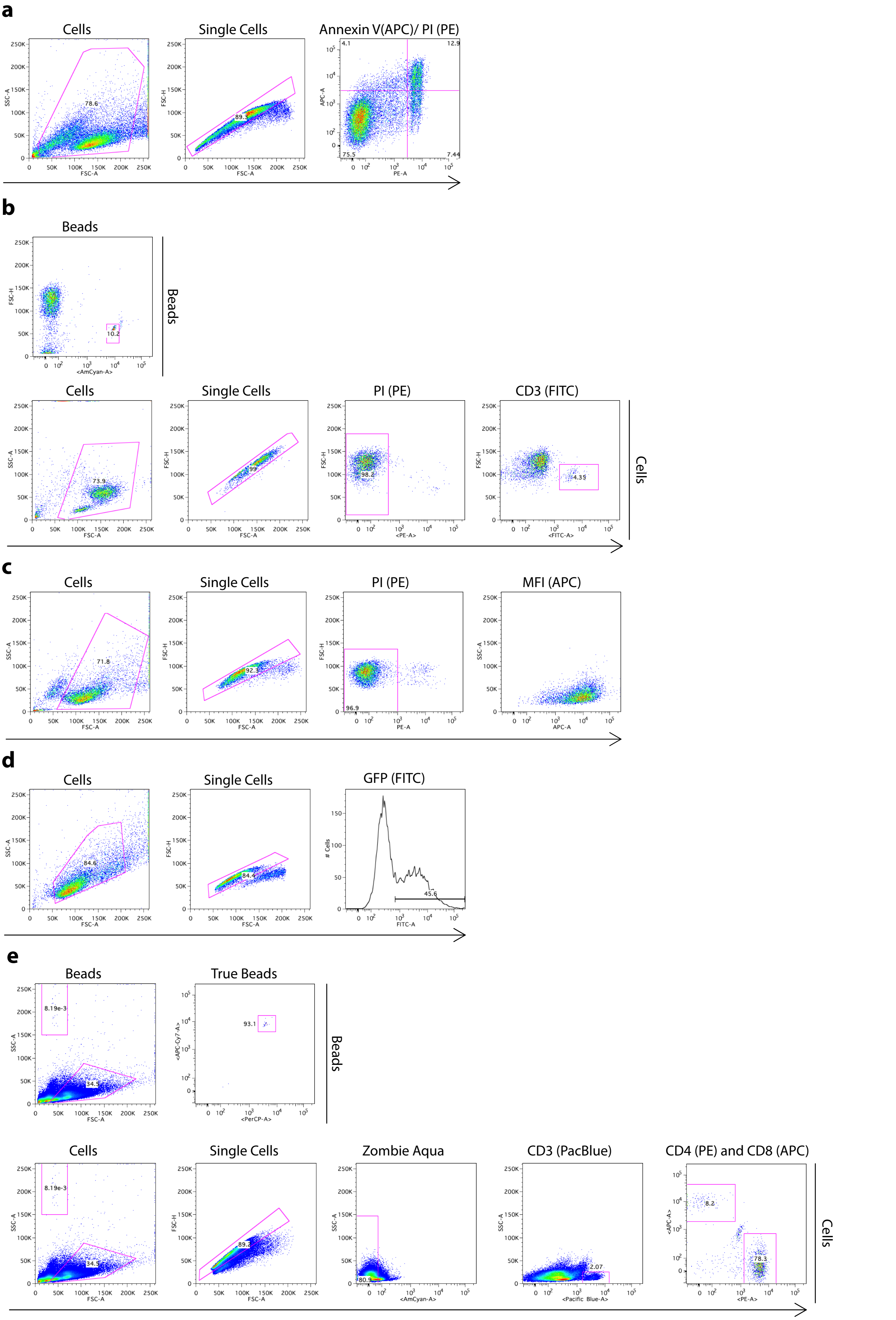

Supplement: Supplementary file 1 — Additional file 1: Additional Figures 1–4 and Additional Figure Legends. [file 12943_2022_1641_MOESM1_ESM.pdf]
