# Supplementary material for: Neomorphic DNA-binding enables tumor-specific therapeutic gene expression in fusion-addicted childhood sarcoma
Source: Mol Cancer. 2022 Oct 13;21:199. doi: 10.1186/s12943-022-01641-6 (PMC9558418; doi:10.1186/s12943-022-01641-6)
Supplement: Supplementary file 3 — Additional file 3: Additional Methods. [file 12943_2022_1641_MOESM3_ESM.docx]

## Additional Methods

### Provenience of cell lines, and cell culture conditions

Human cell lines 293T, HeLa, Hep-G2, MHH-ES1, PA-TU-8988T, RD-ES, RH30, SK-N-MC, and U2-OS were obtained from the German Collection of Microorganisms and Cell Cultures (DSMZ) (Braunschweig, Germany)*.* The human A-673 and MRC-5 cell lines were purchased from the American Type Culture Collection (ATCC)*.* TC-71*,* TC-106 was obtained from the Children’s Oncology Group (COG). RH4 and RD were kindly gifted by R. Kappler (Munich, Germany). A-673/TR/shEF1 and A-673/TR/shctrl were kindly gifted by J. Alonso (Madrid, Spain)^1^. Primary human umbilical vein endothelial cells (HUVEC) were kindly provided by S. Massberg (Munich, Germany). All cell lines were cultivated in a humidified atmosphere with 5% CO_2_ at temperature of 37°C. Except for 293T and HUVEC, all cell lines were grown in RPMI-1640 medium containing stable L-glutamine and sodium bicarbonate (Sigma-Aldrich) supplemented with 10% tetracycline-free fetal bovine serum (FCS) (Sigma-Aldrich). Unless specified otherwise, 293T were grown in DMEM supplemented with 10% FCS (Sigma-Aldrich). HUVEC were cultured in Endothelial Cell Growth Medium (Cell Applications, Inc). Cell lines were routinely tested for mycoplasma contamination by nested PCR, and cell line identity was routinely confirmed by STR profiling.

### Analysis of published chromatin-immunoprecipitation and sequencing (ChIP-seq) and RNA-sequencing (RNA-seq) data

Raw data of published ChIP-seq experiments of EF1 in Ewing sarcoma cell lines A-673 and SK-N-MC were retrieved from the European Nucleotide Archive (Samples used: SRR1593960, SRR1593966, SRR1593985, SRR1593991)^2^. Data analysis was performed following the ENCODE Transcription Factor and Histone ChIP-seq processing pipeline^3^: First, quality of raw data was assessed with FastQC^4^. Reads were aligned to the human reference genome (*hg38)* using *bowtie2*^5^. Alignments that were unmapped, not primarily aligned, failing platform checks, duplicates, or aligned with c <30 were removed using s*amtools view* and *picard MarkDuplicates*^6,7^. Peaks were called on by *SPP* from the bioconda package *phantompeakqualtools* using npeak = 30000^3^. To determine the peaks reproducible for both A-673 and SK-N-MC, the Irreproducibility Discovery Rate (IDR) framework was applied with a threshold of 5% using the bioconda package *idr^8^.* Genomic locations (*hg38*) of GGAA-msats, defined as 4 GGAA repeats on either strand, were obtained using the *locate* function of *seqkit^9,10^.* GGAA-msats overlapping or within 100bp from each other, were merged with the *merge* function of the bedtools suite^11^. By intersecting the EF1 peaks with the GGAA-msat locations, EF1-bound msats were defined, and their genomic annotation and closest RefSeq TSS were obtained using HOMER^11,12^. For analysis of previously published RNA-seq experiments from the same study were retrieved from the European Nucleotide Archive (samples used: SRR1594020, SRR1594021, SRR1594024, SRR1594025). *salmon* was used to quantify transcript abundance^13^. Differentially expressed genes after 48h of shRNA-mediated knockdown of EF1 compared to control cell lines without knockdown were determined by use of the R package *DeSeq2*^14^. For a more conservative estimation for genes with low read counts, log2FCs were shrunk using *apeglm* within the *lcfShrink* wrapper function^15^.

### Cloning and plasmid preparation

All cloning was designed on benchling.com and performed using standard restriction-ligation approaches. Plasmid integrity was verified using Sanger-sequencing and agarose gel electrophoresis. All primers and sequences of synthetic linear DNA molecules used for cloning can be found in **Additional Table 4**. To create firefly luciferase reporter plasmids, we inserted a PacI restriction site into the multiple cloning site (MCS) of pGL4.17 [*luc2*/Neo] (Promega) upstream of the EcoRV restriction site using annealed oligonucleotides (Eurofins Genomics) following a KpnI-EcoRV restriction digest. We then inserted double stranded synthetic DNA fragments composed of 17, 21, or 25 GGAA-repeats upstream of the recently described minimal activity promoter YB-TATA (Genscript) into the newly designed MCS by PacI-EcoRV restriction digest^16^. The constitutive promoter of the human elongation factor 1-alpha gene was cloned from pOTTC407 (Addgene #60058) and inserted by PacI-EcoRV restriction digest.

Suicide gene therapeutic plasmids were sequentially created using the EF.CMV.RFP backbone (Addgene #17619) by replacing the EF1 promoter by the synthesized 25-GGAA-YB-TATA fragment (Genscript) followed by a firefly *luciferase* (cloned from pcDNA3-luciferase (Addgene #18964)) or *HSV-TKSR39* (cloned from pET23d:HSVTK-SR39 (kindly provided by Margaret Black, Washington State University)) gene or both P2A-fused genes, and a WPRE (cloned from pLenti CMV GFP Puro (658-5) (Addgene #17448)) all flanked by epigenetic insulator sequences by repetitive restriction digests (KflI-EcoRI, PacI-EcoR1, SpeI-EcoRI, SalI-EcoRI, Bsu36I-AsiSI). RFP was replaced by the puromycin-resistance gene (cloned from pLenti CMV GFP Puro (658-5)), respectively, using a BamHI-NsiI restriction digest after additional insertion of a NsiI digestion site by PCR. Lastly, the full construct was transferred to the p156RRL-sinPPT backbone derived from pLenti CMV GFP Puro (658-5) after ClaI-KpnI restriction. To this end, we inserted a new MCS harboring an additional AgeI restriction site using annealed oligonucleotides (Eurofins Genomics) and inserted the construct by AgeI-NsiI restriction digest resulting in *pLenti_25_LT_Puro*. For creation of all other plasmids, insulator sequences were removed by PCR. For IL-15 and XCL1 expressing plasmids, the *IL-15*-P2A-*XCL1* gene was synthesized by Genscript and inserted by SpeI-SalI digest. To allow better secretion, a mouse IgV signal peptide was inserted right after the start codon of IL-15^17^. To reduce the toxicity of XCL1 on *E. coli*, a synthetic intron (113 nt) was included^18^. For genomic reporter assays, the microsatellite-YB-TATA fragment was excised by PacI-SpeI double restriction digest and replaced by a synthetic, unique gRNA binding sequence from the *luciferase* gene fused to YB-TATA. Subsequently, the *HSV-TKSR39*-P2A-*luciferase* transgene was replaced by eGFP cloned from pLenti CMV GFP Puro (658-5) by SpeI-SalI restriction digest. gRNA binding prediction was performed using the CRISPR tool of benchling.com. For ARMS, the GGAA-microsatellite was replaced by genomic or synthetic alk-super-enhancer (SE) sequences fused to the YB-TATA promoter by double restriction digest (SbfI-SpeI)^19^. Full plasmid maps can be retrieved from the authors upon request. All ligation reactions were performed at room temperature (RT) for 30 min using T4 ligase (NEB). Bacterial transformation was performed following standard protocols using Stellar competent cells (Takara). Mini- and MidiPreps (Macherey-Nagel) were performed according to the manufacturer’s instructions.

### Extraction of total DNA and RNA, reverse transcription, and quantitative Real-Time PCR (qRT-PCR)

Total DNA extraction was performed using NucleoSpin® Tissue mini kit (Macherey-Nagel, Germany). RNA was extracted using NucleoSpin® RNA mini kit (Macherey-Nagel, Germany) including a 15 min DNAse-treatment, and reversely transcribed using the High-Capacity cDNA Reverse Transcription Kit (Applied Biosystems). qRT-PCRs were performed in a final volume of 15 µl using SYBR™ Select Master Mix (Applied Biosystems). All primer sequences used for qRT-PCR are listed in **Additional Table 4**. Cycling conditions were as follows: 95°C for 10 min (initial denaturation), then 50 cycles at 95°C for 10 sec (denaturation) and 60°C for 1 minute (annealing, elongation and detection).

### Lentivirus production and concentration

Lentivirus production was performed by polyethylenimine (PEI)-mediated transfection of adherent 293T cells as previously described^20^. In short, 24 h before transfection, 5.3×10^5^ cells were seeded per well (6-well) in 2 ml fully supplemented DMEM containing 10% FCS. On day of transfection 1020 ng lentiviral transfer plasmid, 680 ng of pCD/NL-BH*DDD (Addgene plasmid # 17531) and either 340 ng of pCEF-VSV-G (Addgene plasmid # 41792) or 680ng of 2.2 (Addgene plasmid # 34885) were mixed in 100 µl final volume of Opti-MEM (Gibco). 15.12 µl of PEI Max Transfection Grade Linear Polyethylenimine Hydrochloride MW 40.000 (Polysciences) (1 mg/ml) were diluted in a final volume of 100 µl Opti-MEM in a separate tube. After 5 min of individual incubation, the PEI mix was added to the plasmid mix and mixed by pipetting. The resulting PEI-plasmid mix was incubated for 5 min. In the meantime, the medium of the previously seeded 293T cells was replaced by 2 ml of, either DMEM for transfections using VSV-G virus, or UltraCULTURE medium (Lonza) for transfections with 2.2. After incubation, the PEI-plasmid mix was added to the medium dropwise. The medium was replaced by fresh DMEM or UltraCULTURE 16 h after transfection. After an additional 48 h, the supernatant containing the viral particles was collected. To remove cellular debris, the harvested medium was centrifuged for 5 min at 1000 g and the resulting supernatant filtered through a syringe filter (0.45 µm pore size, CA membrane). When high viral titers were needed (i.e. for *in viv*o experiments), virus production was upscaled on 150 mm dishes and the viral supernatant was concentrated using polyethylene glycol-based precipitation. To this end, 3 parts of supernatant were mixed with 1 part of custom-made lentivirus concentrator solution (40% w/w PEG8000, 1.337 M NaCl, 2.7 mM KCl, 8 mM Na_2_HPO_4_ und 2 mM KH_2_PO_4_) and incubated at 4°C overnight on a roller mixer. After 24 h, the mixture was centrifuged at 2000 g for 1 h, the supernatant was discarded and the resulting pellets were resuspended in PBS. Viral titers were calculated based on flow cytometry (FACSCanto™ II, BD) results or qPCR as described previously^20^.

### *In vitro* lentiviral transduction

Cell lines were transduced using a standard MOI of 2 (U2-OS reference) by adding equal amounts of virus-containing, filtered supernatants as previously described^21^. Where indicated, cells were selected using puromycin (Invivogen) at the minimum concentration necessary for complete cell death determined for each cell line individually.

### Production of antibody-coated lentiviruses

2.2 pseudotyped lentivirus was produced as outlined above. Viral supernatants or concentrated viral particles were mixed with the respective antibody (5 µg/ml for *in vitro* and 15 µg/ml for *in vivo* experiments) and incubated for 5 min on RT.

### Clustered regularly interspaced short palindromic repeats (CRISPR) knock-in

For genomic reporter assays, A-673 cells were lentivirally transduced with a *GFP* reporter gene downstream of the minimal promoter YB-TATA^16^ and a unique guide-RNA binding site. After puromycin-selection, the transduced cells were single cell cloned and clones harboring a single copy of the reporter construct were identified using genomic qPCR as previously described^20^. Two clones were selected and 4×10^4^ cells in 100 µl complete medium were CRISPRed using reverse lipofection technique (Lipofectamine CRISPRMAX, LifeTechnologies) of 30 nM ALT-R CAS9 nucleoproteins (IDT) and 25 nM 25 GGAA-repeat HDR templates (50 bp homology arms each, IDT) in a 96-well plate. After 48 h, the medium was changed and the cells were expanded. GFP-positive cells were single-cell-sorted into a 96-well plate using a FACSAria™ II (BD). After expansion of the individual GFP-positive clones, cells were lysed and DNA was isolated as described above. The correct insertion of 25 GGAA-repeats upstream of the YB-TATA promoter was confirmed by Sanger-sequencing of the PCR-amplified genomic region. Cells were imaged using a Zeiss Axiovert 25 microscope and the Zeiss AxioVision (Release 4.9.1 SP2) software. Images were overlaid and contrast-enhanced using Adobe Photoshop (Adobe). Fluorescence intensity was measured by flow cytometry (FACSCanto™ II, BD).

### Dual-luciferase reporter assays

5×10^4^ cells were seeded per well (24-well) 24 h prior to transfection. Cells were co-transfected with the plasmid pGL4.17 containing a YB-TATA-based minimal activity promoter and enhancer sequences (GGAA-msats of various lengths, genomic or synthetic alk-SE sequences) and pRL *Renilla* Luciferase Control Vector (Promega) (plasmid mass ratio of pGL4.17:pRL = 100:1). Where indicated, reporter plasmids were co-transfected with a plasmid containing either *EWSR1-FLI1* cDNA (pCDH-CMV-E/F1-puro) or a defective mutant (or pCDH-CMV-E/F1_R2L2-puro^22^). Furthermore, in experiments with A673/TR/shEF1 and A673/TR/shCtrl medium was supplemented with doxycycline 1 µg/ml (Sigma-Aldrich) to achieve shRNA-mediated knockdown of EF1. Transfections were performed using PEI MAX (Polysciences) for all cell lines apart from Jurkat and RD for which Lipofectamine LTX was used. 12 h after transfection, the medium was replaced with fresh RPMI containing 10% FCS. After 36 h, cells were lysed and luminescence measured using a dual-luciferase assay kit (Beetle-Juice Luciferase assay firefly and *Renilla*-Juice Luciferase Assay, PJK GmbH). Firefly luciferase induced luminescence was normalized on *Renilla* luciferase luminescence and the resulting ratios were normalized to that of the empty control plasmid condition.

### Western blot

Western blots were performed as previously described^23^. For preparation of protein lysates, 3×10^5^ *pLenti_25_LT_Puro-*transduced and selected cells were seeded per well (6-well). After 48 h, medium was removed, cells were washed with 1 ml of PBS and lysed by adding 100 µl of lysis buffer containing 150 mM NaCl, 0.1% Triton X-100, 50 mM Tris-HCl at pH 8.0 supplemented with cOmplete, Mini, EDTA-free Protease Inhibitor Cocktail (Roche). Detection of specific bands for firefly luciferase or GAPDH was performed using a HRP-conjugated monoclonal Anti-Luciferase antibody (sc-74548 HRP, 1:2,000, Santa-Cruz) and a HRP-conjugated, monoclonal murine Anti-GAPDH antibody (HRP-60004, 1:50,000, Proteintech).

### Cell viability assays

5×10³ (1×10^4^ for TC-106) *pLenti_25_LT_Puro-*transduced and selected cells were seeded in 90 µL medium per well (96-well). After 24 h, ganciclovir (GCV) was added for final concentrations ranging from 0.01 µM to 50 µM with 0.05% dimethyl sulfoxide (DMSO) in all conditions. 72 h after the addition of GCV, cell viability was assessed using a resazurin-based readout system^24^. Relative fluorescence units of treated wells were background corrected and normalized to vehicle controls.

### Apoptosis assays

5×10^4^ of *pLenti_25_LT_Puro-*transduced and selected cells were seeded per well (24-well). 24 h later, GCV was added for final concentrations of 0.4 µM. After 72 h, apoptosis was analyzed by Annexin V/PI staining (APC Annexin V Apoptosis Detection Kit with PI, Biolegend) and flow cytometry using a FACSCanto™ II (BD) cytometer. An example of the gating strategy is found in **Additional Fig. 4a**.

### IL-15 and XCL1 ELISAs

3×10^5^ *pLenti_25_IX_Puro-*transduced or wildtype cells were seeded in 0.5 ml per well (12-well) and incubated for 72 h in RPMI 1640 containing 10% FCS. Supernatants were harvested and stored at -20°C until further use. IL-15 and XCL1 levels were quantified using human IL-15 Duoset ELISA (RnD) and human XCL1 Duoset ELISA (RnD) according to the manufacturer’s protocol. Cytokine levels were calculated by constructing a 4-parameter logistic regression model based on standard measurements for each plate using the R package *drc*^25^.

### In vitro T cell migration assays

Migration assays were performed using 96-Transwell plates with 3 µm pore size (Corning). 225 µl conditioned medium was transferred into the lower chamber, before 1×10^6^ freshly isolated Peripheral blood mononuclear cells (PBMC) of healthy donors were loaded onto the membrane in 70 µl complete medium. After 4 h, the transwell insert was removed and the cells in the lower chamber were collected, stained for CD3 (Biolegend), analyzed by flow cytometry (FACSCanto™ II, BD) and quantified using Precision Count Beads (Biolegend). An example of the gating strategy can be found in **Additional Fig. 4b**.

### Microarray analysis

To identify genes encoding membrane proteins that could serve as potential binding points for targeted transduction of EwS cells, we took advantage of a gene expression previously described data set of publicly available microarray data (Affymetrix HG-U133Plus2.0) consisting of 928 normal human tissue samples and 50 EwS samples^26^. Robust Multiarray Average (RMA) normalization and calculation of expression measures was performed using the function *just.RMA* of the bioconductor package *affy*^27^. Genes that were statistically significantly overexpressed in EwS compared to every other tissue with a fold change of at least 2 were identified using the R package *limma* using a false discovery rate (FDR) cutoff of 5%^28^. Multiple testing was accounted for using the Benjamini-Hochberg procedure. Accession codes of samples used in the analysis can be found in **Additional Table 5**.

### Analysis of protein expression by indirect flow cytometry

2×10^5^ cells were seeded per well (12-well) 24 h prior to analysis. Cells were harvested using trypsin and washed in PBS twice. Subsequently, cells were then stained with the primary antibody for the indicated antigens (0.25 µg per 1×10^6^ cells) for 30 min at RT. Cells were washed three times with PBS, before the secondary antibody (0.375 µg per 1×10^6^ cells) was applied for 30 min at RT. After three additional washing cycles, stained cells were co-stained with propidium iodide (PI) solution and analyzed on a FACSCanto™ II (BD) cytometer. An example of the gating strategy is found in **Additional Fig. 4c**. The following antibodies were used: CD99 (3B2/TA8, Biolegend), FAT4 (NBP1-78381, Novus Biologicals), GPR64 (purified using Mouse TCS Antibody Purification Kit (ab128749, abcam) from OAM6#93 (PTA-5704, ATCC), GD2 (TAB-731, Creative Biolabs), Mouse IgG2b Isotype Control (#02-6300, Invitrogen), Rabbit IgG Isotype Control (#02-6102), Goat anti-Rabbit IgG (H+L) Cross-Adsorbed Secondary Antibody, APC (A-10931, Invitrogen), Goat anti-Mouse IgG (H+L) Cross-Adsorbed Secondary Antibody, APC (A-865, Invitrogen).

### Tissue microarrays and evaluation for immunoreactivity

Formalin-fixed samples tissue microarrays of EwS-samples and normal tissues were stained for

GPR64 after antigen retrieval using Target Retrieval Solution (Fa.Agilent Technologies, S1699) with anti-GPR64 (purified using Mouse TCS Antibody Purification Kit (ab128749, abcam) from OAM6#93 (PTA-5704, ATCC) with a concentration of 40 µg/ml for 1 h at RT. For signal detection the MACH 3 Mouse HRP Polymer Detection system was employed according to manufacturer’s protocol using DAB+ (Fa.Agilent Technologies, K3468). Slides were counter-stained with Hematoxylin Gill`s Formula (Fa.Vector, H-3401). Signal intensities were evaluated by two blinded resident pathologists using a semi-quantitative score in analogy to the previously described Immune Reactive Score (IRS)^26^.

### Evaluation of targeted transduction *in vitro*

5×10^4^ cells were plated in 400 µl per well (24-well). 24 h later, 100 µl of unconcentrated, 2.2 pseudotyped lentiviral supernatant were mixed with 0.5 µg antibody and added to each well (final concentration of antibody: 1 µg/µl). After 24 h, supernatants were removed and cells were incubated for an additional 24 h. Cells were then harvested and fluorescence was analyzed by flow cytometry. An example of the gating strategy is found in **Additional Fig. 4d**.

### *In vitro* therapy assays

5×10³ cells were seeded in 90 µl of medium per well (96-well). After 24 h, equal amounts of concentrated lentivirus (approx. 1000 transducing units, TU) were added to each well. After additional 24 h, GCV was added for a final concentration of 20 µM. Cell viability was assessed by a resazurin-based assay 72 h after the addition of GCV.

### Luciferase-based evaluation of promoter activity and specificity *in vivo*

To assess promoter-dependent gene expression in various non-EwS tissues 2×10^7^ TU (transducing units) of VSV-G-pseudotyped virus produced with *pLenti_25_LT* or *pLenti_CMV_LG* were injected in 200 µl PBS intraperitoneally into NSG mice (NOD.Cg-Prkdc^SCID^Il2rg^tm1Wjl^/SzJ, Charles River Laboratories). 7 days later, luminescence was measured on an IVIS-100 (Perkin-Elmer) imaging system after intraperitoneal injection of 3 mg D-luciferin.

### Mouse xenograft experiments

For subcutaneous xenograft experiments, six- to eight-week old NSG mice were subcutaneously injected into the flank with 2×10^6^ wildtype or pre-transduced A-673 or RD-ES EwS cells in Cultrex Basement Membrane Extract (R&D Systems) to enhance tumor formation. Tumor growth was measured three times a week using a caliper. Tumor volumes were calculated using the following formula: V = L × W^2^ / 2, where V is tumor volume, L is largest diameter and W smallest diameter. For intraperitoneal engraftment, 2×10^6^ luciferase-expressing A-673 cells were intraperitoneally injected. Animal experiments were approved by the government of Upper Bavaria and conducted in accordance with ARRIVE guidelines, recommendations of the European Community (86/609/EEC), and UKCCCR (guidelines for the welfare and use of animals in cancer research).

### *In vivo* tumor transduction

For the evaluation of antibody-directed transduction of subcutaneous xenografts (**Fig. 2f**), 0.5×10^6^ TU of 2.2 or VSV-G pseudotyped virus was injected intratumorally in 100 µl PBS containing 15 µg/ml antibody where indicated. For therapeutic transduction of subcutaneous xenografts (**Fig. 2g**), 1×10^7^ TU (*pLenti_25_LT)* of 2.2 pseudotyped lentivirus was injected intratumorally in 100 µl PBS containing 15 µg/ml anti-GPR64 antibody twice per week once the tumor had reached a mean diameter of 5 mm. For *pLenti_25_IX*, we injected 0.5×10^7^ TU of 2.2 pseudotyped lentivirus intratumorally in 100 µl PBS containing 15 µg/ml anti-GPR64 antibody on 3 consecutive days once tumors reached a mean diameter of 5mm. For therapeutic transduction of intraperitoneal tumor masses, 2×10^7^ TU of 2.2 pseudotyped lentivirus was injected intraperitoneally in 200 µl PBS containing 15 µg/ml anti-GPR64 antibody on three consecutive days (day 3 to 5), three days after tumor inoculation. A second round of three consecutive viral injections was performed on day 13 to 15. Bioluminescence imaging after intraperitoneal injection of 3 mg D-luciferin was performed on days 6, 12 and 19.

### Oral Valganciclovir (VGCV) administration

For the treatment of *pLenti_25_LT* or *pLenti_25_TK* (pre-)transduced xenografts, VGCV (0.5 mg/ml) was administered orally *ad libidum* by addition to the drinking water. To mitigate any adverse taste, 5% sucrose (Carl Roth) was added as well. 5% sucrose containing drinking water served as a control where indicated.

### Human T cell transduction

Human PBMC were freshly isolated by density gradient centrifugation using Biocoll 1.077 g/ml (Bio&Sell). CD3^+^ T cells were sorted using CD3 magnetic beads (Miltenyi) and stimulated using T cell TransAct (Miltenyi) according to the manufacturer's protocol. T cells were maintained in T cell medium containing 10% FCS (Sigma Alderich), 100 U ml^-1^ penicillin (Biochrom), and 100 μg ml^-1^ streptomycin (Biochrom), 1% L-Glutamine (Sigma Alderich), 1% Sodium Pyruvate (Sigma Alderich), 1% non-essential amino acids (NEAA, Sigma Alderich), 84 IU/ml human IL-15 (Miltenyi) and 500 IU/ml human IL-7 (Mitenyi). For GFP expression, 0.5×10^6^ cells were lentivirally transduced using VSV-G pseudotyped virus (pLenti CMV GFP Puro (658-5)) 24h post stimulation. T cells were expanded for 12 days before subsequent use.

### T cell transfer and *in vivo* migration assay

1×10^7^ GFP-transduced T cells were intravenously injected into the tail vein of tumor bearing NSG mice. T cell migration into the subcutaneously growing xenografts was assessed 5 days after T cell transfer by flow cytometry analysis. To this end, tumors were harvested, weighed, minced and digested in Collagenase/DNAse (1 mg/ml and 100 Kunitz/ml) for 30 min. at 37°C. Single cell suspensions were prepared by manual tissue homogenization through 70 µm and 30 µm cell strainers (Miltenyi). Cells were stained for human CD3, CD4 and CD8 (Biolegend). Dead cells were excluded using the Zombie Aqua Fixable Viablity Staining Kit (Miltenyi). CD3^+^ CD4^+^ and CD3^+^ CD8^+^ human T cells were counted using Precision Count beads (Biolegend). An example of the gating strategy is found in **Additional Fig. 4e**.

### Statistical analysis

Data was analyzed using R (R version 4.1.2, R Foundation for Statistical Computing, Vienna, Austria). Where not otherwise specified, the statistical significance of differences between two experimental groups were tested using the two-tailed Wilcoxon Rank Sum / Mann-Whitney test with the Holm–Bonferroni method to account for multiple comparisons; * :p <= 0.05, **: p <= 0.01, ***: p <= 0.001, ****: p <= 0.0001.

## Supplementary References:

1. Carrillo J, García-Aragoncillo E, Azorín D, et al. Cholecystokinin Down-Regulation by RNA Interference Impairs Ewing Tumor Growth. *Clin Cancer Res*. 2007;13(8):2429-2440. doi:10.1158/1078-0432.CCR-06-1762

2. Riggi N, Knoechel B, Gillespie SM, et al. EWS-FLI1 Utilizes Divergent Chromatin Remodeling Mechanisms to Directly Activate or Repress Enhancer Elements in Ewing Sarcoma. *Cancer Cell*. 2014;26(5):668-681. doi:10.1016/j.ccell.2014.10.004

3. Landt SG, Marinov GK, Kundaje A, et al. ChIP-seq guidelines and practices of the ENCODE and modENCODE consortia. *Genome Res*. 2012;22(9):1813-1831. doi:10.1101/gr.136184.111

4. Babraham Bioinformatics - FastQC A Quality Control tool for High Throughput Sequence Data. Accessed December 30, 2021. https://www.bioinformatics.babraham.ac.uk/projects/fastqc/

5. Langmead B, Salzberg SL. Fast gapped-read alignment with Bowtie 2. *Nat Methods*. 2012;9(4):357-359. doi:10.1038/nmeth.1923

6. Danecek P, Bonfield JK, Liddle J, et al. Twelve years of SAMtools and BCFtools. *GigaScience*. 2021;10(2):giab008. doi:10.1093/gigascience/giab008

7. Picard Tools - By Broad Institute. Accessed December 30, 2021. https://broadinstitute.github.io/picard/

8. *Irreproducible Discovery Rate (IDR)*. Kundaje Lab; 2021. Accessed December 30, 2021. https://github.com/kundajelab/idr

9. Gangwal K, Sankar S, Hollenhorst PC, et al. Microsatellites as EWS/FLI response elements in Ewing’s sarcoma. *Proc Natl Acad Sci*. 2008;105(29):10149-10154. doi:10.1073/pnas.0801073105

10. Shen W, Le S, Li Y, Hu F. SeqKit: A Cross-Platform and Ultrafast Toolkit for FASTA/Q File Manipulation. *PLOS ONE*. 2016;11(10):e0163962. doi:10.1371/journal.pone.0163962

11. Quinlan AR, Hall IM. BEDTools: a flexible suite of utilities for comparing genomic features. *Bioinformatics*. 2010;26(6):841-842. doi:10.1093/bioinformatics/btq033

12. Heinz S, Benner C, Spann N, et al. Simple Combinations of Lineage-Determining Transcription Factors Prime cis-Regulatory Elements Required for Macrophage and B Cell Identities. *Mol Cell*. 2010;38(4):576-589. doi:10.1016/j.molcel.2010.05.004

13. Patro R, Duggal G, Love MI, Irizarry RA, Kingsford C. Salmon provides fast and bias-aware quantification of transcript expression. *Nat Methods*. 2017;14(4):417-419. doi:10.1038/nmeth.4197

14. Love MI, Huber W, Anders S. Moderated estimation of fold change and dispersion for RNA-seq data with DESeq2. *Genome Biol*. 2014;15(12):550. doi:10.1186/s13059-014-0550-8

15. Zhu A, Ibrahim JG, Love MI. Heavy-tailed prior distributions for sequence count data: removing the noise and preserving large differences. *Bioinformatics*. 2019;35(12):2084-2092. doi:10.1093/bioinformatics/bty895

16. Ede C, Chen X, Lin MY, Chen YY. Quantitative Analyses of Core Promoters Enable Precise Engineering of Regulated Gene Expression in Mammalian Cells. *ACS Synth Biol*. 2016;5(5):395-404. doi:10.1021/acssynbio.5b00266

17. Meazza R, Gaggero A, Neglia F, et al. Expression of two interleukin-15 mRNA isoforms in human tumors does not correlate with secretion: role of different signal peptides. *Eur J Immunol*. 1997;27(5):1049-1054. doi:10.1002/eji.1830270502

18. Tikhonov MV, Maksimenko OG, Georgiev PG, Korobko IV. Optimal artificial mini-introns for transgenic expression in the cells of mice and hamsters. *Mol Biol*. 2017;51(4):592-595. doi:10.1134/S0026893317040173

19. Gryder BE, Yohe ME, Chou HC, et al. PAX3–FOXO1 Establishes Myogenic Super Enhancers and Confers BET Bromodomain Vulnerability. *Cancer Discov*. 2017;7(8):884-899. doi:10.1158/2159-8290.CD-16-1297

20. Kuroda H, Marino MP, Kutner RH, Reiser J. Production of Lentiviral Vectors in Protein‐free Media. *Curr Protoc Cell Biol*. 2011;50(1). doi:10.1002/0471143030.cb2608s50

21. Knott MML, Cidre-Aranaz F. Ewing Sarcoma-Specific (Re)expression Models. In: Cidre-Aranaz F, G. P. Grünewald T, eds. *Ewing Sarcoma : Methods and Protocols*. Methods in Molecular Biology. Springer US; 2021:119-138. doi:10.1007/978-1-0716-1020-6_9

22. Bailly RA, Bosselut R, Zucman J, et al. DNA-binding and transcriptional activation properties of the EWS-FLI-1 fusion protein resulting from the t(11;22) translocation in Ewing sarcoma. *Mol Cell Biol*. Published online May 1994. doi:10.1128/mcb.14.5.3230-3241.1994

23. Marchetto A, Romero-Pérez L. Western Blot Analysis in Ewing Sarcoma. In: Cidre-Aranaz F, G. P. Grünewald T, eds. *Ewing Sarcoma : Methods and Protocols*. Methods in Molecular Biology. Springer US; 2021:15-25. doi:10.1007/978-1-0716-1020-6_2

24. Musa J, Cidre-Aranaz F. Drug Screening by Resazurin Colorimetry in Ewing Sarcoma. In: Cidre-Aranaz F, G. P. Grünewald T, eds. *Ewing Sarcoma : Methods and Protocols*. Methods in Molecular Biology. Springer US; 2021:159-166. doi:10.1007/978-1-0716-1020-6_12

25. Ritz C, Baty F, Streibig JC, Gerhard D. Dose-Response Analysis Using R. Xia Y, ed. *PLOS ONE*. 2015;10(12):e0146021. doi:10.1371/journal.pone.0146021

26. Baldauf MC, Orth MF, Dallmayer M, et al. Robust diagnosis of Ewing sarcoma by immunohistochemical detection of super-enhancer-driven EWSR1-ETS targets. *Oncotarget*. 2017;9(2):1587-1601. doi:10.18632/oncotarget.20098

27. Gautier L, Cope L, Bolstad BM, Irizarry RA. affy—analysis of Affymetrix GeneChip data at the probe level. *Bioinformatics*. 2004;20(3):307-315. doi:10.1093/bioinformatics/btg405

28. Ritchie ME, Phipson B, Wu D, et al. limma powers differential expression analyses for RNA-sequencing and microarray studies. *Nucleic Acids Res*. 2015;43(7):e47. doi:10.1093/nar/gkv007
